# Estimating the number of Canadians suffering from fecal incontinence using pooled prevalence data from meta-analysis

**DOI:** 10.3389/fgstr.2024.1398102

**Published:** 2024-09-03

**Authors:** Ava Oliaei, Dean Elterman, Salar Sadri, Eric Zimmerman, Padina Pezeshki, Bilal Chughtai, Hamid Sadri

**Affiliations:** ^1^ Department of Interdisciplinary Studies, McMaster University, Hamilton, ON, Canada; ^2^ Department of Urology, University of Toronto, Toronto, ON, Canada; ^3^ Department of Law, University of Toronto, Toronto, ON, Canada; ^4^ Department of Art and Science, McGill University, Montreal, QC, Canada; ^5^ Department of Clinical and Medical Affairs, Medtronic, Brampton, ON, Canada; ^6^ Department of Urology, Weill Cornell Medical College, New York, NY, United States; ^7^ Department of Health Economics and Outcomes Research, Medtronic, Brampton, ON, Canada

**Keywords:** systematic review, fecal incontinence, prevalence, meta-analysis, population estimate

## Abstract

**Background and aim:**

Fecal incontinence (FI) is defined as the unintended loss of solid or liquid stool. FI adversely affects the patient’s quality of life. However, due to stigma, lack of awareness, and underdiagnosis, there is a notable gap in the knowledge regarding its prevalence. This study aimed to conduct a systematic review and meta-analysis of published literature reporting on FI prevalence and estimate the number of people afflicted by FI.

**Methods:**

A systematic review was conducted following the PRISMA 2020 guidelines, using the Embase, MEDLINE, CINHAL, and PubMed databases to identify relevant publications in the English language. Two reviewers independently screened the articles and extracted data. The reference sections and content of the review papers were also evaluated. Thirty-two articles were selected and included. A meta-analysis of proportions was performed using RStudio software. A sub-analysis was conducted to account for the variation between sample population age groups to minimize heterogeneity. The pooled prevalence was extrapolated to the Canadian population and a sample of ten densely populated countries to estimate the number of people affected by FI.

**Results:**

The Mean pooled FI prevalence in men and women was 7% (95% CI: 6-9%) and 10% (95% CI: 8-12%), respectively. The sub-analysis mean pooled prevalence of FI in men and women was 8% (95% CI: 6-10%) and 10% (95% CI: 8-12%), respectively. The authors estimate that between 1 and 1.5 million Canadians and 320 to 500 million people in the ten most populous countries suffer from FI.

**Conclusion:**

Fecal incontinence is a prevalent underdiagnosed condition requiring appropriate and timely treatment to improve a patient’s quality of life.

## Introduction

Fecal incontinence (FI) is defined as the unintended loss of solid or liquid stool (1). FI substantially burdens an individual’s life, and patients often report lower quality of life (QoL) (2). Despite the significant burden, FI continues to be under-reported and undertreated, which stems from the stigma surrounding FI and the general lack of knowledge and awareness (3). This unawareness may impact the patient-physician interaction during the consultation, as many patients are embarrassed to discuss their symptoms with the healthcare provider or believe the symptoms are part of normal aging (4). Additionally, clinicians are often reluctant to ask about incontinence, thereby perhaps compounding the problem (5).

More than 50% of patients with FI are estimated not to seek treatment (6). A survey of individuals seeking gastroenterology consultations showed that 84% of FI patients did not proactively inform the specialist during the consultation (2). Furthermore, the diagnosis of FI is hindered by its heterogeneity and subjective nature. The differences in the etiology and pathophysiology of FI deter an objective assessment, wherein stigma and wording of the question can influence the results (7). As such, determining FI’s prevalence has proven challenging. Understandably, due to underreporting and underdiagnosis, no extensive population survey has been conducted on FI in Canada to date. This study aimed to conduct a systematic review and meta-analysis of published literature that reports on FI prevalence (primary outcomes)to better understand the prevalence of FI in Canada and estimate the number of people suffering from FI (secondary outcomes) to assist health policymakers in making informed decisions addressing FI.

## Methods

### Search strategy

**Figure 1:**
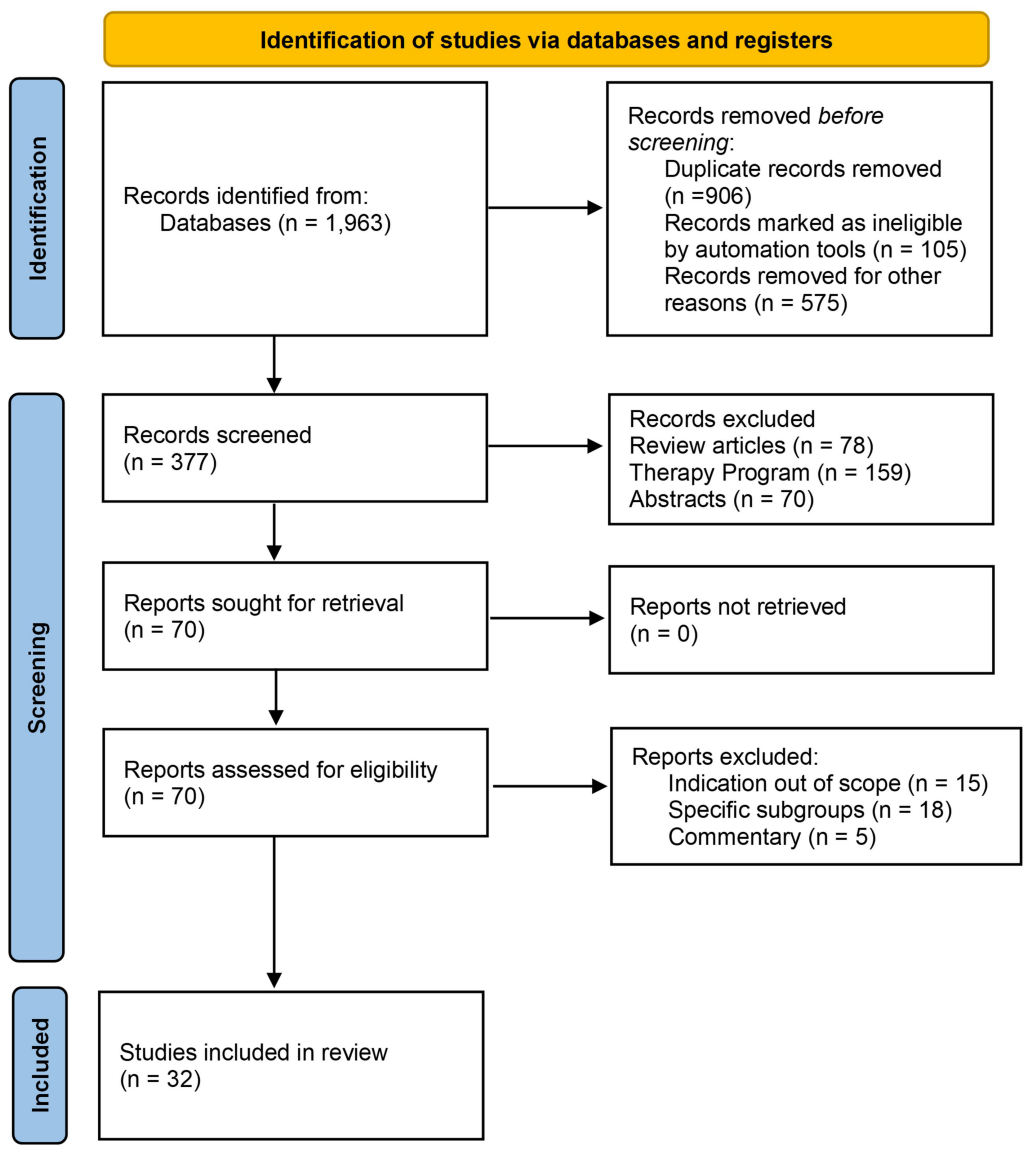
PRISMA literature search screening and selection flow-chart for fecal incontinence.

We conducted a systematic review according to the “ Preferred Reporting Items for Systematic Reviews and Meta-Analyses “ (PRISMA) 2020 guidelines (**Figure 1**, **Supplementary Table S1**) (8).

We used the Embase, MEDLINE, CINHAL, and PubMed databases until December 2023. The search strategy focused on the terms “fecal incontinence” and “accidental bowel leakage” in conjunction with “prevalence” and “epidemiology.” The texts, tables, and graphs of the articles were thoroughly examined for data extraction. Additionally, the reference section and content of the review papers were evaluated. Two sets of reviewers (HS, AO and SS, EZ) independently screened and assessed the titles and abstracts of the identified articles, and studies were included in the analysis based on the following criteria:

### Inclusion criteria

Scope: Fecal incontinence prevalence and epidemiology study

Population: Adult patients with an FI diagnosis

Type of study: Epidemiology studies

Publications: Full article published in the English language

### Exclusion criteria

Out of scope: Other pathophysiology, such as post-surgical and post-partum retention

Publication type: Conference abstracts, review articles, letters, commentary, non-human studies, and non-English publications

Inconsistencies in identifying relevant studies were deliberated to reach an agreement. A pre-specified EXCEL sheet (Microsoft, Redmond, CA, USA) was used for data extraction, including the study year, country, reported prevalence data, number of patients, and age brackets.

### Critical appraisal for risk of bias

Two sets of reviewers (HS & AO and SS & EZ) evaluated the risk of bias in the included studies using the Newcastle-Ottawa Scale (NOS). The NOS was designed to assess the quality of non-randomized studies in meta-analyses (9). The NOS uses a star system to assess various aspects of the study, including the selection of study groups, comparability between groups, and choosing the outcome of interest in cohort studies. Each criterion can be given one star within the “selection” and “outcome” categories, whereas comparability can receive up to two stars. Individual study quality and risk of bias were assessed using the approach described by Ribeiro et al. (10). Studies with a total score of ≥7, 6, or ≤5 were considered to have a low, medium, and high risk of bias, respectively.

### Analysis

A meta-analysis of proportions was conducted on the FI prevalence data from the included publications using the “Meta-prop” function in the RStudio software (Posit, Boston, MA, USA). Forest plots were created using the “forest” function of the software. Furthermore, to account for the variation in age ranges within the study sample populations and to minimize heterogeneity in the meta-analysis, subgroup analyses were performed for FI prevalence in men and women, excluding studies that did not report data for all ages above 20 years.

### Canadian and international estimates

To determine the number of patients with FI in Canada, we applied the pooled prevalence derived from the meta-analysis to Canadian population data stratified by age, sex, and province using the current Statistics Canada demographic data (**Supplementary Table S2**) (11). Additionally, the pooled prevalence was applied to the latest population estimate in the ten most populous countries worldwide to estimate the number of people suffering from FI. The methodology and characteristics of the included studies are summarized in the **Supplementary Material** (**Supplementary Table S3**). All data are available in the text and **Supplementary Material**. Details are available upon request from the corresponding author.

## Results

### Search strategy

Thirty-two studies were included in this analysis. Thirteen were from North America (12–24), one from South America (25), ten from Europe (2, 26–34), two from the Middle East (35, 36), three from Asia (37–39), and Australia (40–42). The sample size of included articles ranges from a minimum of 128 to a maximum of 42,796. The Median and Mean sample sizes were 749 and 3,398, respectively. Twenty studies provided data on both sexes, and 12 studies were only on females (**Table 1**). All studies were cross-sectional, and the surveys were administered online or in person. Only three studies were longitudinal surveys (22, 24, 25).

**Table 1:** Studies that reported the prevalence of fecal incontinence (percentage by age bracket).

Citation	Country	Sex	N	Prevalence by age brackets (%)												Total
				20-24	25-29	30-34	35-39	40-44	45-49	50-54	55-59	60-64	65-69	70-74	>75	
Alimohammadian (2014) (35)	Iran	F	800					18		18.6		17.2		21		18.4
Bener (2008) (36)	Qatar	F	596					10.4								10.4
Bharucha (2005) (12)	USA	F	2,800	7.3		11.9		17.3		21.7		20		21.1		18.1
Boreham (2005) (13)	USA	F	457	28.4												28.4
Botlero (2011) (40)	Australia	F	442				21.5		15.9		21.1		37.6		25.2	20.7
Brown (2012) (14)	USA	F	5,817						15.6		21.7		19		23.1	18.8
Damon (2006) (2)	France	F	366	5.6				7.9				10				7.5
		M	340	1.4				2.5				4.2				2.4
Demir (2017) (26)	Turkey	F	236									10.2				10.2
		M	128									9.4				9.4
Ditah (2014) (15)	USA	F	7,511	3.		4.5		9.8			14.5			17		8.4
		M	7,248	3		5.2		7.2			9.0			15.0		8.4
Edwards (2001) (27)	UK	F	1,099										4.0			4.0
		M	980										1.0			1.0
Goode (2005) (16)	USA	F	499										11.6			11.6
		M	501										12.4			12.4
Halland (2013) (41)	Australia	F	4,815												10.4	10.4
Horng (2014) (37)	Taiwan	F	1,370										9.3			9.3
		M	1,345										6.5			6.5
Lim (2014) (38)	Singapore	F	201	4.7												4.7
		M	180	4.7												4.7
Lopez-Colombo (2012) (17)	Mexico	F	305	4.3												4.3
		M	195	5.1												5.1
Meinds (2017) (28)	Netherlands	F	680	11			9		5.5		8.1		5.8			7.9
		M	579	11			9		5.5		8.1		5.8			7.9
Melville (2005) (18)	USA	F	3,444			3.5		3.5		7.5		12		12.5		7.2
		F	42,696	9.2	13.2				16.1				18.3			14.4
Menees (2018) (19)	USA	M	29,116	9.2	13.2				16.1				18.3%			14.4%
Ng (2015) (42)	Australia	F	258	12.1												12.1
		M	138	12.1												12.1
Nygaard (2008) (20)	USA	F	1,961	2.9				9.9%				14.4-21.6				9
Parés (2011) (34)	Spain	F	332	12												12
		M	186	8.6												8.6%
Perry (2002) (29)	UK	F	5,483					3.6		4.3		5.8		7.7-11.7		5.7
		M	4,633					4.1		5.7		6.9		7.8- 11.6		6.2
Quander (2005) (21)	USA	F	3,720										6.9		11.1-19.5	9.6
		M	2,379										6.9		11.1 - 19.5	9.6
Rey (2010) (22)	USA	F	749							17.7						17.7
		M	764							12.6						12.6
Rømmen (2012)	Norway	F	20,391			1.7		1.5		2.2		3.8		5.4-7.5		3.0
Roslani (2014) (39)	Malaysia	F	760	5.1	6				9.7				18.7			8.3
		M	240	5.1	6				9.7				18.7			8.3
Santacruz (2017) (33)	Spain	F	415	1		4.3		10.3		11.8		7.1				7.8
		M	415	1		4.3		10.3		11.8		7.1				6.2
S-t Hove (2009) (31)	Netherlands	F	1,869						9.7	14.5	13.4	14.2	16.9	22.2		14.2
Tamanini (2016) (25)	Brazil	F	864									13.2				13.2
		M	481									8.3				8.3
van Meegdenburg (2018) (32)	Netherlands	F	680	7.9												7.9
		M	579	7.9												7.9
Whitehead (2009) (23)	USA	F	2,229	3.0		3.5		9.5			14.5			15.3		8.9
		M	2,079	2.6		6.5		8.5			9			15.5		7.7
Wu (2014) (24)	USA	F	7,924	2.6		4.3		8.8		11		16.5		14.3 - 21		9.4

F, female; M, male; N, number.

### Pooled data

**Figure 2:**
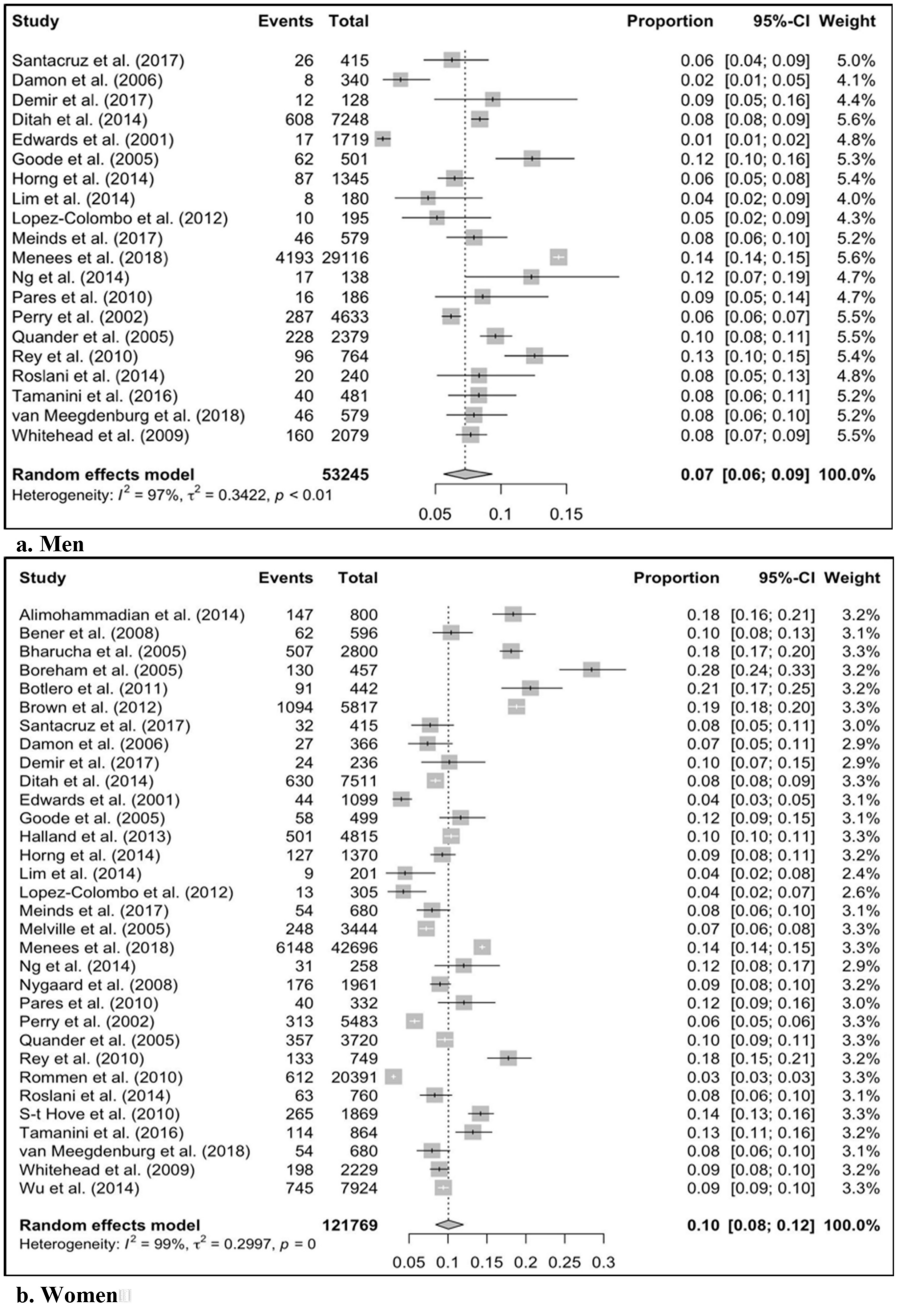
The pooled prevalence for fecal incontinence in men and women. CI, Confidence Interval.

The Mean pooled prevalence of FI in men and women was 7% (95% CI: 6–9%) and 10% (95% CI: 8–12%), respectively (**Figure 2**). The sub-analysis mean pooled prevalence for FI in men and women was 8% (95% CI: 6–10%) and 10% (95% CI: 8–12%), respectively (**Supplementary Figure S1**).

### Assessing the risk of bias

All the included studies had a low risk of bias. The mean NOS score of the studies was 7.7 out of 9, with a range of 7.5 - 9 (**Supplementary Table S4**).

### Extrapolating to the Canadian population

The pooled meta-analysis data indicated that 6–10% of men and 8–12% of women over 20 years of age have experienced FI. Thus, we estimate that between 1 and 1.5 million people suffer from FI in Canada (**Table 2**).

**Table 2:** Estimated number of people in Canada with fecal incontinence by province.

Province	Number of People (1,000)	
	F	M
Canada	930 - 1,550	900 - 1,500
Alberta	136 - 204	102 - 170
British Columbia	172 - 258	126 0 210
Manitoba	42.4 - 63.6	31.2 - 52
New Brunswick	26.4 - 39.6	19.5 - 32.5
Newfound land	17.6 - 26.4	12.6 - 21
Northwest Territories	1.3 - 1.9	1.0 - 1.7
Nova Scotia	33.6 - 50.4	24 - 40
Nunavut	0.9 - 1.4	0.7 - 1.2
Ontario	488 - 732	354- 590
Prince Edwards Island	5.4 - 8.1	3.9 - 6.6
Quebec	276 - 52.8	204 - 340
Saskatchewan	35.2 - 52.8	26.4 - 44
Yukon	1.4 - 2.1	1- 1.7

FI, fecal incontinence; F, female; M, male.

### International estimates

Moreover, we extrapolated the pooled prevalence of FI to the ten most populous countries worldwide to assess FI’s global impact of FI. We estimated that between 320 million and 500 million people suffer from FI in these countries (**Table 3**).

**Table 3:** Estimates of fecal incontinence in the ten most populous countries.

Country	Population (Mil.)			The number of people with Incontinence (Mil.)			
				FI			
	Total	F	M	F		M	
				min	max	min	max
India	1,428.63	691.78	736.85	55.34	83.01	44.21	73.69
China	1,425.67	698.5	727.17	55.88	83.82	43.63	72.72
USA	340	171.7	168.3	13.74	20.6	10.1	16.83
Indonesia	277.53	137.83	139.71	11.03	16.54	8.38	13.97
Pakistan	240.49	119.23	121.26	9.54	14.31	7.28	12.13
Nigeria	223.8	110.67	113.13	8.85	13.28	6.79	11.31
Brazil	216.42	110.18	106.24	8.81	13.22	6.37	10.62
Bangladesh	172.95	87.28	85.67	6.98	10.47	5.14	8.57
Russia	144.44	77.36	67.09	6.19	9.28	4.03	6.71
Mexico	128.46	65.81	62.64	5.27	7.9	3.76	6.26
Total	4,598.39	2270.34	2328.06	181.63	272.44	139.68	232.81

FI, fecal incontinence; F, female; M, male; min, minimum; max, maximum; Mil, million.

Source: 2023 population estimates. Population pyramid. Accessed December 25th, 2023, from https://www.populationpyramid.net.

### Treatment options

Considering the grave impact of FI on a patient’s QoL, treatment with FI can profoundly affect a patient’s well-being. Various pharmacological and non-pharmacological treatments are available for FI, including behavioral therapies (e.g., dietary modifications and bowel retraining), physical therapies (e.g., biofeedback), anti-diarrheal medications, padding, and mechanical inserts (e.g., plugs) (43). Surgical options (e.g., artificial bowel sphincter) are considered for unresponsive patients who lack long-term success (7).

Another viable option is sacral neuromodulation (SNM), a minimally invasive procedure that restores neural communication between the brain and bowel, making it an effective therapy for FI. This therapy uses a small lead surgically implanted adjacent to the sacral nerve and stimulates the nerve using electrical currents originating from an implanted pulse generator. Initially, patients undergo a peripheral nerve evaluation (PNE), an indicator of treatment response. In responsive patients who receive a permanent implant, 92% achieve significant symptom remission after six months (43–45). The aforementioned surgical treatments for FI have up to 40% morbidity, while SNM complications occur in up to 20% of patients and primarily involve localized pain at the site of the PNE or implant (7, 43).

## Discussion

This systematic review and meta-analysis pooled international data to estimate fecal incontinence prevalence. Our results demonstrated a 7% and 10% pooled prevalence of FI in men and women, respectively. To our knowledge, this is the first study attempting to infer sex-stratified FI based on prevalence data of FI in individuals aged > 20 years in Canada. The Canadian FI prevalence by sex and province was estimated by overlaying the pooled prevalence in the Canadian provincial population data.

Our results are comparable with those of Whitehead et al., who reported that 17.6% of Canadians had experienced at least one FI symptom, although they did not specify the symptom or differentiate the prevalence between the sexes (23). The Canadian Incontinence Foundation estimates that 5% of the non-hospitalized population, 1% of those under 65 years, and 4%–7% of those over 65 years are afflicted by FI, while the data from international surveys on FI range from 7% to 12% (28). Our study results were aligned with other studies that reported a prevalence of 9.1% and 7.6% for women and men, respectively (19).

Some of the data differences can be attributed to the variations among clinical definitions of FI (e.g., frequency and elapsed time of involuntary stool) and questionnaires on FI. Whitehead found that, while 16% of their sample reported at least one FI symptom, 7% and 3.3% met the Rome III and Rome IV criteria, respectively (23). Another variation between studies was the time intervals, spanning from a week to a lifetime, which could result in different prevalence. The 2015 US National Gastrointestinal Survey found that 14.4% of the respondents had lifetime experience with FI. However, only 4.7% of the patients had FI within the last seven days (19).

In this study, we did not conduct an age-adjusted meta-analysis because comprehensive data for each age group were unavailable. However, consistent with previous studies, we observed a trend of increasing FI rate with age (19).

Furthermore, sub-analyses using published data for individuals over 20 years of age demonstrated no change in the FI rate. We expected a lower pooled proportion value because studies with the same sample populations and greater risk for such conditions were excluded. Menees et al. reported a higher lifetime prevalence for those over 20 years of age, and Horng et al. reported a lower one-year prevalence for those over 65 (19, 37).

The current study focused on sex-specific FI prevalence to increase physicians’ and patients’ awareness. The results of this study showed a slightly higher prevalence of FI in women than in men. In contrast, other studies have not found a significant difference between the sexes (23). Women are likely to be more susceptible to FI because of factors such as childbirth trauma and being prone to irritable bowel syndrome (44). It is also possible that our results have been skewed due to the higher willingness of women to report FI symptoms compared to men (46).

Due to the stigma and unawareness, FI is underreported and underdiagnosed. Additionally, the subjective nature of assessment tools used for FI diagnosis would cause substantial variability in diagnosis, making assessing the actual prevalence of FI through a large-scale nationwide study impractical (4).

The ramifications of FI underreporting and a lack of awareness in clinical practice are multifold. First, eligible patients may not have received timely and appropriate care. In addition, many patients with FI may not be referred to specialized centers for more advanced and cost-effective therapies (e.g., SNM) (47). The high prevalence of FI emphasizes the need to diagnose patients using standardized criteria proactively. Particularly in Canada, with a growing aging population, we expect an increase in FI prevalence, an increasing financial burden, and a declining QoL (19).

Additionally, this study considered data from the English language publications. Although this might be considered a limitation and potentially skew the data, studies have shown the impact of social constructs, beliefs, language, and wording in the diagnosis and scoring tools. Consequently, we believe these inclusion criteria are justified and aligned with the study goal.

This study estimated the number of patients with FI by sex in Canada. Based on the quintuple healthcare framework, achieving optimal outcomes at a reasonable cost with flexibility for professional judgment requires appropriate and timely planning that reflects demographic changes (48). These data are critical for FI, an underreported and stigmatized disease (49).

Patients with unmonitored FI are likely to suffer from comorbidities, increasing the burden on the individual and the healthcare system (50). Thus, interventions with effective treatments and strategies can help reduce the burden of healthcare resource utilization. Furthermore, measures that improve efficiency by reducing healthcare and hospital resources or long-term complications may be implemented to help leaders and hospital managers optimize their offerings within budget constraints. In this study, we provide an example of an opportunity in which the prevalence of the disease in the general population is presented. Similar opportunities may aid healthcare leaders and decision-makers in optimizing care delivery based on patient outcomes and values. Collectively, and over time, such policies can improve the efficiency of healthcare delivery more comprehensively for different therapy areas (51).

## Limitations

The results of this study should be considered within the limitations. First, the methodology for data collection, sampling, clinical measures, and timelines varied across studies. The heterogeneity of the data required a random-effects meta-analysis model in our study. The varied methodology might account for some of the reported differences in the prevalence data. Varying clinical measures between studies led to different item scores, wording, and response formats.

Rømmen et al. employed a more stringent criterion, considering an FI diagnosis only if participants reported experiencing at least one occurrence of FI per week over the past month, resulting in an overall prevalence of 3% (30). In contrast, the study by Lim et al., which utilized in-person interviews for data collection, reported an overall prevalence of 4.7%, while the study by Bharucha et al., which employed a mailed questionnaire, found an overall prevalence of 18.1% (12, 38). This divergence in findings may be attributed to individuals experiencing embarrassment or hesitation to discuss their symptoms during face-to-face interviews.

The analyzed studies were also heterogeneous in data collection using self-administered questionnaires, in-person reviews, remote (online and telephone) surveys, and variable settings (e.g., clinics, hospitals, or private residences). The sampling methods and populations differed (e.g., random sampling or the general population). Further research on the current prevalence of FI in the Canadian population is warranted. Widespread population-based surveys that utilize consistent diagnostic criteria and sampling methodologies, as well as electronic health record data to estimate the number of patients with FI, could substantiate our findings, better estimate SNM candidates, and raise awareness of the importance of this treatment.

## Conclusion

Fecal incontinence is prevalent among men and women, but it is underdiagnosed and underreported, and requires more attention to improve patient quality of life.

## Data Availability

The original contributions presented in the study are included in the article/**Supplementary Material**. Further inquiries can be directed to the corresponding author.
