# Supplementary figures and images for: Estimating the number of Canadians suffering from fecal incontinence using pooled prevalence data from meta-analysis
Source: Front Gastroenterol (Lausanne). 2024 Sep 3;3:1398102. doi: 10.3389/fgstr.2024.1398102 (PMC12952408; doi:10.3389/fgstr.2024.1398102)

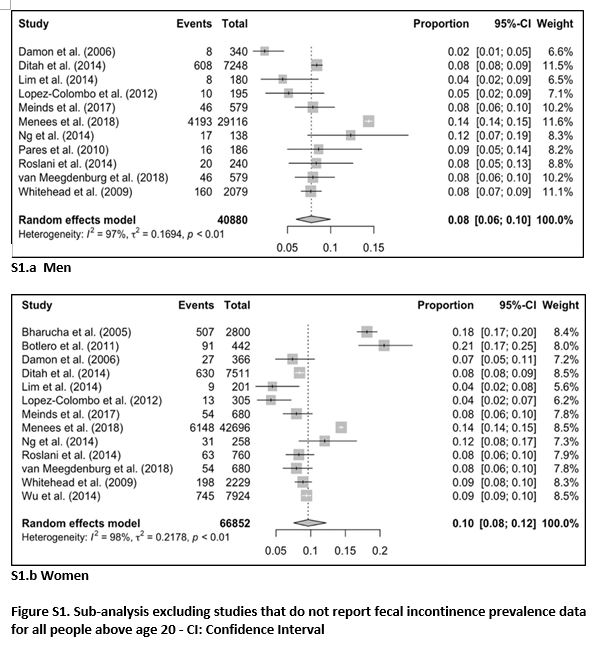

Supplement: Supplementary file 1 [file Image1.jpg]
