# Supplementary material for: Estimating the number of Canadians suffering from fecal incontinence using pooled prevalence data from meta-analysis
Source: Front Gastroenterol (Lausanne). 2024 Sep 3;3:1398102. doi: 10.3389/fgstr.2024.1398102 (PMC12952408; doi:10.3389/fgstr.2024.1398102)
Supplement: Supplementary file 2 [file Table1.docx]

**Table S1- PRISMA checklist**

| **Section/topic** | **#** | **Checklist item** | **Reported on page #** |
| --- | --- | --- | --- |
| **TITLE** | | |  |
| Title | 1 | Identify the report as a systematic review, meta-analysis, or both. | Page 1 |
| **ABSTRACT** | | |  |
| Structured summary | 2 | Provide a structured summary including, as applicable: background; objectives; data sources; study eligibility criteria, participants, and interventions; study appraisal and synthesis methods; results; limitations; conclusions and implications of key findings; systematic review registration number. | Page 1 |
| **INTRODUCTION** | | |  |
| Rationale | 3 | Describe the rationale for the review in the context of what is already known. | Page 2 |
| Objectives | 4 | Provide an explicit statement of questions being addressed concerning participants, interventions, comparisons, outcomes, and study design (PICOS). | Page 2 |
| **METHODS** | | |  |
| Protocol and registration | 5 | Indicate if a review protocol exists, if and where it can be accessed (e.g., Web address), and, if available, provide registration information, including registration number. | - |
| Eligibility criteria | 6 | Specify study characteristics (e.g., PICOS, length of follow-up) and report characteristics (e.g., years considered, language, publication status) used as criteria for eligibility, giving rationale. | Page 3 |
| Information sources | 7 | Describe all information sources (e.g., databases with coverage dates, contact with study authors to identify additional studies) in the search and the date last searched. | Page 3 |
| Search | 8 | Present a complete electronic search strategy for at least one database, including any limits used so that it could be repeated. | Page 3 |
| Study selection | 9 | State the process for selecting studies (i.e., screening, eligibility, included in the systematic review, and, if applicable, included in the meta-analysis). | Page 4 |
| Data collection process | 10 | Describe the data extraction method from reports (e.g., piloted forms, independently, in duplicate) and any processes for obtaining and confirming investigator data. | Page 4 |
| Data items | 11 | List and define all variables for which data were sought (e.g., PICOS, funding sources) and any assumptions and simplifications made. | Page 4 |
| Risk of bias in individual studies | 12 | Describe methods used for assessing the risk of bias in individual studies (including specification of whether this was done at the study or outcome level) and how this information will be used in any data synthesis. | Page 4 |
| Summary measures | 13 | State the principal summary measures (e.g., risk ratio, the difference in means). | Page 4 |
| Synthesis of results | 14 | Describe the methods of handling data and combining results of studies, if done, including measures of consistency (e.g., I^2^) for each meta-analysis. | Page 4 |
| Risk of bias across studies | 15 | Specify any assessment of the risk of bias that may affect the cumulative evidence (e.g., publication bias, selective reporting within studies). | Page 4 |
| Additional analyses | 16 | Describe methods of additional analyses (e.g., sensitivity or subgroup analyses, meta-regression), if done, indicating which were pre-specified. | Page 4 |
| **RESULTS** | | |  |
| Study selection | 17 | Give the number of studies screened, assessed for eligibility, and included in the review, with reasons for exclusions at each stage, ideally with a flow diagram. | Page 5 |
| Study characteristics | 18 | For each study, present characteristics for which data were extracted (e.g., study size, PICOS, follow-up period) and provide the citations. | Page 5 |
| Risk of bias within studies | 19 | Present data on the risk of bias of each study and, if available, any outcome level assessment (see item 12). | Page 5 Table S2 |
| Results of individual studies | 20 | For all outcomes considered (benefits or harms), present for each study: (a) simple summary data for each intervention group, (b) effect estimates and confidence intervals, ideally with a forest plot. | Page 6 Table S3 |
| Synthesis of results | 21 | Present results of each meta-analysis, including confidence intervals and consistency measures. | Page 5 Figure 2 |
| Risk of bias across studies | 22 | Present results of any assessment of the risk of bias across studies. | Table S2 |
| Additional analysis | 23 | Give results of additional analyses, if done (e.g., sensitivity or subgroup analyses, meta-regression. | Figure S1 |
| **DISCUSSION** | | |  |
| Summary of evidence | 24 | Summarize the main findings, including the strength of evidence for each main outcome; consider their relevance to key groups (e.g., healthcare providers, users, and policymakers). | Page 7 |
| Limitations | 25 | Discuss limitations at the study and outcome levels (e.g., risk of bias) and review level (e.g., incomplete retrieval of identified research, reporting bias). | Page 11 |
| Conclusions | 26 | Provide a general interpretation of the results in the context of other evidence and implications for future research. | Page 11 |
| **FUNDING** | | |  |
| Funding | 27 | Describe funding sources for the systematic review and other support (e.g., data supply); the role of funders for the systematic review. | Title page |
