# Supplementary material for: Estimating the number of Canadians suffering from fecal incontinence using pooled prevalence data from meta-analysis
Source: Front Gastroenterol (Lausanne). 2024 Sep 3;3:1398102. doi: 10.3389/fgstr.2024.1398102 (PMC12952408; doi:10.3389/fgstr.2024.1398102)
Supplement: Supplementary file 3 [file Table2.docx]

| **Province** | **NL** | | **PEI** | | **NS** | | **NB** | | **QC** | | **ON** | | **MN** | | **SK** | | **AL** | | **BC** | | **YT** | | **NT** | | **NU** | |
| --- | --- | --- | --- | --- | --- | --- | --- | --- | --- | --- | --- | --- | --- | --- | --- | --- | --- | --- | --- | --- | --- | --- | --- | --- | --- | --- |
| **Age Bracket** | **M** | **F** | **M** | **F** | **M** | **F** | **M** | **F** | **M** | **F** | **M** | **F** | **M** | **F** | **M** | **F** | **M** | **F** | **M** | **F** | **M** | **F** | **M** | **F** | **M** | **F** |
| 20 to 24 | 16,024 | 13,985 | 7,539 | 6,222 | 35,980 | 31,836 | 25,507 | 22,876 | 252,047 | 234,618 | 552,722 | 504,641 | 56,110 | 49,464 | 40,413 | 36,534 | 145,314 | 135,804 | 176,835 | 166,828 | 1,464 | 1,276 | 1,542 | 1,552 | 1,603 | 1,542 |
| 25 to 29 | 14,724 | 13,684 | 7,334 | 5,977 | 36,487 | 34,142 | 25,208 | 22,753 | 288,244 | 265,084 | 584,381 | 541,489 | 51,520 | 46,895 | 39,408 | 35,935 | 158,507 | 149,256 | 192,867 | 179,924 | 1,549 | 1,383 | 2,069 | 1,632 | 1,603 | 1,592 |
| 30 to 34 | 14,792 | 14,664 | 5,117 | 5,098 | 33,379 | 34,030 | 24,259 | 24,048 | 297,251 | 279,080 | 561,758 | 541,969 | 50,447 | 49,474 | 41,900 | 40,674 | 180,344 | 173,495 | 202,831 | 197,776 | 1,760 | 1,738 | 1,948 | 1,839 | 1,661 | 1,666 |
| 35 to 39 | 14,544 | 15,419 | 4,902 | 5,069 | 31,370 | 32,364 | 24,428 | 24,534 | 288,460 | 278,109 | 523,249 | 518,982 | 49,314 | 49,602 | 45,719 | 44,669 | 187,600 | 182,291 | 195,575 | 192,658 | 1,837 | 1,952 | 1,754 | 1,637 | 1,429 | 1,382 |
| 40 to 44 | 14,701 | 16,211 | 4,870 | 5,145 | 28,651 | 31,338 | 24,442 | 24,557 | 304,874 | 293,906 | 465,479 | 488,136 | 45,514 | 45,088 | 41,606 | 39,695 | 171,267 | 169,363 | 172,516 | 176,880 | 1,673 | 1,711 | 1,698 | 1,743 | 1,322 | 1,238 |
| 45 to 49 | 16,244 | 17,439 | 4,975 | 5,254 | 28,883 | 31,245 | 25,752 | 25,815 | 278,340 | 268,656 | 446,204 | 473,902 | 42,238 | 40,998 | 35,281 | 34,241 | 150,903 | 147,799 | 157,756 | 165,904 | 1,326 | 1,527 | 1,300 | 1,506 | 1,104 | 1,001 |
| 50 to 54 | 19,007 | 20,042 | 5,228 | 5,539 | 31,331 | 33,134 | 27,114 | 26,839 | 267,088 | 261,585 | 468,251 | 484,244 | 39,955 | 40,442 | 33,113 | 32,081 | 140,208 | 134,755 | 166,750 | 178,528 | 1,452 | 1,400 | 1,557 | 1,539 | 1,296 | 1,149 |
| 55 to 59 | 20,619 | 21,598 | 5,682 | 5,857 | 36,603 | 38,207 | 30,334 | 30,699 | 304,628 | 301,784 | 514,199 | 523,312 | 41,626 | 43,086 | 36,333 | 35,247 | 136,874 | 136,151 | 177,243 | 186,828 | 1,440 | 1,449 | 1,803 | 1,552 | 1,031 | 940 |
| 60 to 64 | 20,942 | 21,643 | 5,631 | 6,038 | 38,099 | 40,126 | 30,933 | 31,834 | 313,629 | 316,409 | 492,464 | 512,395 | 42,512 | 43,363 | 37,780 | 37,216 | 135,357 | 137,989 | 178,732 | 189,801 | 1,547 | 1,619 | 1,535 | 1,162 | 589 | 663 |
| 65 to 69 | 19,646 | 20,697 | 5,100 | 5,638 | 33,934 | 36,358 | 28,638 | 29,932 | 275,806 | 283,907 | 408,422 | 447,008 | 36,572 | 38,235 | 33,505 | 33,598 | 115,042 | 118,362 | 158,714 | 173,501 | 1,384 | 1,286 | 1,016 | 1,062 | 453 | 280 |
| 70 to 74 | 16,963 | 17,817 | 4,616 | 4,922 | 28,195 | 30,895 | 23,511 | 25,644 | 223,514 | 237,084 | 329,862 | 372,866 | 28,620 | 31,255 | 24,731 | 25,382 | 83,054 | 89,219 | 132,672 | 145,626 | 878 | 847 | 660 | 564 | 310 | 235 |
| 75 to 79 | 11,778 | 12,678 | 3,168 | 3,541 | 20,183 | 23,172 | 16,694 | 18,415 | 165,586 | 184,609 | 241,500 | 282,226 | 20,277 | 22,658 | 16,794 | 18,555 | 55,525 | 63,020 | 94,743 | 104,552 | 578 | 500 | 417 | 324 | 160 | 144 |
| 80 to 84 | 6,260 | 7,389 | 1,646 | 2,274 | 11,577 | 14,657 | 9,713 | 11,789 | 98,073 | 121,579 | 149,941 | 192,116 | 12,241 | 15,354 | 10,339 | 13,455 | 33,354 | 41,458 | 57,521 | 67,075 | 281 | 217 | 151 | 156 | 77 | 68 |
| 85 to 89 | 2,971 | 4,015 | 909 | 1,479 | 5,954 | 8,870 | 4,862 | 7,283 | 52,283 | 77,643 | 85,806 | 125,146 | 6,861 | 10,021 | 5,887 | 9,308 | 18,482 | 26,636 | 32,371 | 42,844 | 98 | 136 | 104 | 56 | 30 | 23 |
| 90 to 94 | 995 | 1,817 | 335 | 663 | 2,152 | 4,528 | 1,873 | 3,817 | 22,173 | 44,023 | 35,991 | 67,354 | 2,901 | 5,955 | 2,598 | 5,536 | 7,595 | 13,905 | 13,869 | 22,497 | 58 | 35 | 18 | 40 | 14 | 10 |
| 95 to 99 | 211 | 557 | 83 | 218 | 474 | 1,438 | 366 | 1,232 | 5,050 | 15,295 | 8,591 | 22,531 | 689 | 2,194 | 601 | 1,783 | 1,586 | 4,343 | 2,864 | 7,547 | 13 | 14 | 1 | 10 | 3 | 0 |

**Table S2- Canadian provinces population data by age bracket (August 2023)**

NL: Newfoundland & Labrador, PEI: Prince Edwards Island, NS: Nova Scotia, NB: New Brunswick, QC: Québec, ON: Ontario, MN: Manitoba, AL: Alberta, BC: British Columbia, YT, Yukon, NT: Northwest Territory, NU, Nunavut

**Source: Statistics Canada. Population estimates on July 1st, by age and sex. doi:10.25318/1710000501-ENG.**
