# Supplementary material for: Estimating the number of Canadians suffering from fecal incontinence using pooled prevalence data from meta-analysis
Source: Front Gastroenterol (Lausanne). 2024 Sep 3;3:1398102. doi: 10.3389/fgstr.2024.1398102 (PMC12952408; doi:10.3389/fgstr.2024.1398102)
Supplement: Supplementary file 4 [file Table3.docx]

| **Study** | **Design** | **Method of data collection** | **Setting** | **Population** | **Sampling** | **Diagnostic** | |
| --- | --- | --- | --- | --- | --- | --- | --- |
| **Fecal Incontinence** |  |  |  |  |  | **Tool** | **Criteria** |
| Santacruz 2017 | Cross-Sectional | In-person written survey | Hospital | Non-institutionalized adults | Convenience | CCIS | Any FI occurrence |
| Damon 2006 | Cross-Sectional | Mailed survey | Private Residence | Non-institutionalized adults | Cluster | CCIS | CCIS Score ≥5 |
| Demir 2017 | Cross-Sectional | In-person interview | Outpatient Geriatric Care Center | Patients ≥60 years of age | Convenience | FISI | FI occurrence ≥ once weekly |
| Ditah 2014 | Cross-Sectional | In-person interview | Private Residence | Non-institutionalized adults | Random | FISI | Any FI occurrence in the last month |
| Edwards 2001 | Cross-Sectional | In-person interview | Private Residence | Non-institutionalized ≥65 | Random | N/A | Any FI occurrence |
| Goode 2005 | Cross-Sectional | In-person interview | Private Residence | Patients ≥65 years | Convenience | N/A | Any FI occurrence in the last year |
| Horng 2014 | Cross-Sectional | In-person interview | Private Residence | Non-institutionalized ≥65 | Stratified | N/A | Any FI occurrence in the last year |
| Lim 2014 | Cross-Sectional | In-person interview | Private Residence | Non-institutionalized ≥21 | Random | CFIQ, CCIS | CCIS Score ≥1 |
| Lopez-Colombo 2012 | Cross-Sectional | In-person interview | Private Residence | Non-institutionalized adults | Random | Rome II | Rome II |
| Meinds 2017 | Cross-Sectional | Internet-based survey | Private Residence | Non-institutionalized adults | Random | DeFeC | Rome III |
| Menees 2018 | Cross-Sectional | Telephone survey | Private Residence | Gastrointestinal patients | Random | N/A | Any FI occurrence |
| Ng 2014 | Cross-Sectional | In-person written survey | Primary Health Centers | Adults | Convenience | CCIS | CCIS Score ≥1 |
| Pares 2010 | Cross-Sectional | In-person interview | Primary Health Centers | Adults | Convenience | N/A | Any FI occurrence in the last 4 weeks |
| Perry 2002 | Cross-Sectional | Mailed survey | Private Residence | Non-institutionalized ≥40 | Convenience | N/A | Several FI occurrences per month over last year |
| Quander 2005 | Cross-Sectional | In-person interview | Private Residence | Residents ≥65 | Convenience | N/A | Any FI occurrence over last few months |
| Rey 2010 | Longitudinal | Mailed survey | Private Residence | Patients ≥50 | Stratified | NR | Any FI occurrence |
| Roslani 2014 | Cross-Sectional | Survey | Hospital | Patients ≥18 | Convenience | CCIS | CCIS Score ≥1 |
| Tamanini 2016 | Longitudinal | In-person interview | Private Residence | Non-institutionalized ≥60 | Random | N/A | Any FI occurrence in the last year |
| van Meegdenburg 2018 | Cross-Sectional | Internet-based survey | Private Residence | Non-institutionalized female adults | Random | DeFeC | Rome IV |
| Whitehead 2009 | Cross-Sectional | In-person interview | Private Residence | Non-institutionalized ≥20 | Random | FISI | Any FI occurrence in the last month |
| Alimohammadian 2014 | Cross-Sectional | In-person interview | Private Residence | Non-institutionalized females ≥40 | Cluster | NR | Any FI occurrence |
| Bener 2008 | Cross-Sectional | In-person interview + survey | Primary Health Centers | Non-institutionalized females 39-48 | Random | N/A | Any FI occurrence in the last year |
| Bharucha 2005 | Cross-Sectional | Mailed survey | Private Residence | General + institutionalized non-pregnant, female | Stratified | FICA | Any FI occurrence in the last year |
| Boreham 2005 | Cross-Sectional | In-person written survey | Clinic/hospital | Females 18-65 | Convenience | FISI | Any FI occurrence in the last year |
| Botlero 2011 | Cross-Sectional | Mailed survey | Private Residence | Non-institutionalized females 26-82 | Random | PFDI | Any FI occurrence in the last 3 months |
| Brown 2012 | Cross-Sectional | Internet-based survey | Private Residence | Non-institutionalized females ≥45 | Random | N/A | Any FI occurrence in the last year |
| Halland 2013 | Cross-Sectional | Survey | Primary Health Centers | Females 82-87 | Random | N/A | FI occurrence ≥ once per month over last year |
| Melville et al 2005 | Cross-Sectional | Mailed survey | Private Residence | Females 30-90 | Stratified | N/A | FI occurrence ≥ once monthly |
| Nygaard 2008 | Cross-Sectional | In-person interview | Private Residence | Non-institutionalized, non-pregnant female ≥ 20 | Random | FISI | FI occurrence ≥ once monthly |
| Rommen 2010 | Cross-Sectional | In-person/remote written survey | Private Residence or Research Stations | Non-institutionalized females ≥30 | Convenience | N/A | FI occurrence ≥ once weekly in last month |
| S-t Hove 2010 | Cross-Sectional | Mailed survey | Private Residence | Non-institutionalized females 45-85 | Convenience | DDI | Any FI occurrence in the last year |
| Wu 2014 | Longitudinal | In-person interview | Private Residence | Non-institutionalized, non-pregnant female ≥ 20 | Random | FISI | FI occurrence ≥ once monthly |

**Table S3 – Included study’s methodology**

**CCIS:** Cleveland Clinic Fecal Incontinence Severity Scoring System; **FISI:** Fecal Incontinence Severity Index; **CFIQ:** Comprehensive Fecal Incontinence Questionnaire; **DeFeC:** Groningen Defecation and Fecal Continence; **FICA:** Fecal Incontinence and Constipation Assessment; **PFDI:** Pelvic Floor Disability Index; **DDI:** Defecation distress inventory
