# Supplementary material for: Estimating the number of Canadians suffering from fecal incontinence using pooled prevalence data from meta-analysis
Source: Front Gastroenterol (Lausanne). 2024 Sep 3;3:1398102. doi: 10.3389/fgstr.2024.1398102 (PMC12952408; doi:10.3389/fgstr.2024.1398102)
Supplement: Supplementary file 5 [file Table4.docx]

**Supplementary Table S4: Newcastle-Ottawa Scale of the included studies**

| **Publication** | **Selection** | | | | **Comparability** | **Exposure** | | |
| --- | --- | --- | --- | --- | --- | --- | --- | --- |
|  | **Item 1** | **Item 2** | **Item 3** | **Item 4** | **Item 5** | **Item 6** | **Item 7** | **Item 8** |
| Alimohammadian 2014 | * | * | * | * | * | * | * | * |
| Bener 2008 | * | * | * | * | * | * | * | * |
| Bharucha 2005 | * | * | * | * | * | * | * | * |
| Boreham 2005 | * | / | * | * | * | * | * | * |
| Botlero 2011 | * | * | * | * | * | * | * | * |
| Brown 2012 | * | * | * | * | ** | * | * | * |
| Santacruz 2017 | * | / | * | * | ** | * | * | * |
| Damon 2006 | * | * | * | * | * | * | * | * |
| Demir 2017 | * | / | * | * | * | * | * | * |
| Ditah 2014 | * | * | * | * | * | * | * | * |
| Edwards 2001 | / | * | * | * | * | * | * | * |
| Goode 2005 | * | * | * | * | * | * | * | * |
| Halland 2013 | * | / | * | * | * | * | * | * |
| Horng 2014 | * | * | * | * | * | * | * | * |
| Lim 2014 | * | * | * | * | * | * | * | * |
| Lopez-Colombo 2012 | / | * | * | * | / | * | * | * |
| Meinds 2017 | * | * | * | * | * | * | * | * |
| Melville 2005 | * | * | * | * | ** | * | * | * |
| Menees 2018 | * | * | * | * | * | * | * | * |
| Ng 2015 | * | / | * | * | / | * | * | * |
| Nygaard 2008 | * | * | * | * | * | * | * | * |
| Pares 2011 | * | * | * | * | * | * | * | * |
| Perry 2002 | * | * | * | * | * | * | * | * |
| Quander 2005 | * | * | * | * | / | * | * | * |
| Rey 2010 | * | * | * | * | * | * | * | * |
| Rommen 2012 | * | * | * | * | * | * | * | * |
| Roslani 2014 | * | / | * | * | * | * | * | * |
| S-t Hove 2010 | * | * | * | * | * | * | * | * |
| Tamanini 2016 | * | * | * | * | * | * | * | * |
| vanMeegdenburg 2018 | * | * | * | * | / | * | * | * |
| Whitehead 2009 | * | * | * | * | * | * | * | * |
| Wu 2014 | * | * | * | * | * | * | * | * |

Q1: representativeness of the exposed cohort; Q2: selection of the non-exposed cohort; Q3: ascertainment of exposure; Q4: demonstration that outcome of interest was not present at the start of the study; Q5: comparability of cohorts based on the design or analysis; Q6: assessment of outcome; Q7: was follow-up long enough for outcomes to occur; Q8: adequacy of follow up of cohorts; *: Item get one score; /: Item with no score
